# Aliphatic Poly(Carbonate)s with Acid Responsive Release Mechanisms for Micellar Anti‐Tumor Drug Delivery

**DOI:** 10.1002/marc.202500937

**Published:** 2026-03-13

**Authors:** Adrian V. Hauck, Lutz Nuhn

**Affiliations:** ^1^ Chair of Macromolecular Chemistry, Institute of Functional Materials and Biofabrication, Faculty of Chemistry and Pharmacy, Center of Polymers for Life, Julius‐Maximilians‐Universität‐Würzburg Theodor‐Boveri‐Weg Würzburg Germany

**Keywords:** drug delivery, micelle, nanomedicine, pH‐responsive, polycarbonate

## Abstract

Micellar drug delivery systems have emerged as a versatile platform for improving the solubility, stability, and targeted release of chemo(immuno)therapeutics. This review focuses on micellar formulations that combine two key design principles: the inherent biodegradability of aliphatic poly(carbonate)s and the universal acid‐responsive trigger mechanism. These systems are particularly attractive for tumor therapy, where the acidic microenvironment can be exploited for controlled drug release. We differentiate between two major classes: (i) systems employing acid‐labile linkages for reversible conjugation of pharmaceutically active compounds, and (ii) systems in which micelle disassembly or polymer backbone degradation is governed by acid‐responsive functionalities. Both categories are systematically evaluated according to the chemical motifs enabling acid sensitivity, including oximes, imines, hydrazones, boronate ester, acetals, ketals, and tertiary amines, among others. The review highlights recent advances in synthetic strategies, structure–property relationships, and therapeutic performance, emphasizing how these design elements synergistically enhance drug loading, release kinetics, and biocompatibility. Finally, we discuss current challenges and future directions for translating these smart micellar systems into promising tumor‐targeted (immuno‐)therapeutics.

## Graphical abstract

Micellar systems based on biodegradable aliphatic polycarbonates and acid‐responsive triggers enhance drug solubility, stability, and tumor‐selective release. This review covers micelles with acid‐cleavable drug linkages and those that disassemble via acid‐sensitive functionalities. It highlights key chemistries, structure–property relationships, and therapeutic advances, and outlines remaining challenges and future directions for translating these smart systems into effective tumor‐targeted therapies.

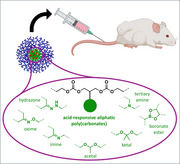

## 1 Introduction

A primary goal in cancer therapy is the elimination of affected cells, either by directly combatting the tumor (chemotherapy) or by reactivating the innate immune system and its mechanisms to recognize and eliminate tumor tissue (immunotherapy) [1, 2, 3]. Although potent drugs have been developed for both strategies, a significant demand remains for improved drug formulations, since tumor therapy is still associated with severe side effects and limited treatment options for many tumor types, largely due to the substantial inter‐ and intra‐tumoral heterogeneity [4, 5, 6]. Besides the development of novel pharmaceutically active compounds, a promising strategy involves optimizing the pharmacokinetics and tissue distribution of existing drugs through the use of advanced drug delivery systems [7, 8]. In this context, the use of nanoparticular structures (1–100(0) nm) has proven beneficial, because their size closely resembles natural (immunogenic) entities such as IgG antibodies (∼15 nm) or IgM antibodies (∼35 nm) that interact with tumors or the immune system [9]. The profound potential of drug delivery systems includes their ability to solubilize often hydrophobic drugs within the body's aqueous environment, thereby, significantly enhancing bioavailability [10]. Moreover, clever chemical design holds the potential to increase blood circulation, enhance the stability of active compounds that are otherwise prone to rapid hydrolytic or enzymatic degradation, enable site‐specific drug release, and facilitate accumulation in tumor tissue through the incorporation of active targeting ligands [11, 12, 13, 14]. To meet all these demands, polymer materials are particularly attractive due to their chemical versatility and ease of modifications toward individual therapeutic needs [15].

Among multiple polymeric nanostructures (nanogels, brush like polymers, nanocomposites, nanospheres, vesicles, nanofibers, dendrimers) polymeric micelles are particularly appealing, as they offer a well‐balanced combination of stability and degradability, helping to prevent the formation of undesired aggregates [16]. Their composition of individual amphiphilic block copolymers facilitates the controlled degradation of micelles following drug delivery, especially when fabricated from biodegradable materials such as aliphatic poly(carbonate)s [17]. The amphiphilic composition of polymeric micelles allows for encapsulating drugs within the micelle's hydrophobic core and the shielding of the active cargo from the environment through the hydrophilic shell [18]. Especially when poly(ethylene glycol) PEG is used as shell, interactions with (lipo‐)proteins in the blood stream can be minimized, contributing to extended circulation times [19]. This represents a significant advantage, especially over lipid nanoparticles (LNPs, used for example in COVID‐19 vaccines), which perform well when administered subcutaneously but tend to be less effective intravenously due to protein adsorption, leading to immune recognition and rapid clearance from the bloodstream [20]. Physical encapsulation of drugs eliminates the need for chemical modification, which may affect potency and reduce biological activity [21, 22]. However, a major drawback of polymeric micelles is their premature drug leakage ahead of reaching the target, primarily, due to limited stability of the formulations [23]. In such case, chemical conjugation of a drug to the carrier can be beneficial, and when implemented with a stimuli‐responsive linker, the drugs potency may be preserved [24]. Beyond covalent drug conjugation via, for example, traceless linkers, stimuli‐responsive functionalities can also be incorporated into a carrier design to induce targeted degradation and enable controlled release of the encapsulated cargo [25]. This approach has the potential to enhance site‐specific drug activity while reducing off‐target toxicities.

The implementation of such triggers typically takes advantage of the distinct environmental conditions at the tumor site compared to healthy tissues [26]. Since tumors are characterized as rapidly proliferating cells, they require an increased oxygen and nutrition supply [27]. In combination with a reduced blood flow due to poor vascularization, this leads to hypoxia (lack of oxygen), which activates genes that induce a changed metabolism in cancer cells [28]. The upregulation of hypoxia induced factor (HIF 1) triggers the expression of glycolytic enzymes and glucose transporters (GLUT1 and GLUT3) [29]. This leads to anaerobic glycolysis in which glycose molecules are metabolized into pyruvates, which are then metabolized into lactic acid, resulting in a reduced pH value (6.2–6.9) within the tumor microenvironment (TME) [30]. This deviation can be used to trigger the decomposition of nanocarriers equipped with acid labile functionalities, while other common triggers in the TME are elevated concentrations of reactive oxygen species (ROS) or glutathione (GSH) levels [31, 32]. Moreover, the lowered pH value can also be exploited in other intracellular compartments like endosomes (pH 6.5–5.0) and lysosomes (pH 4.5–4.0), which is further suitable to address carrier decomposition upon uptake by phagocytosing immune cells like macrophages and dendritic cells (DCs) present in the TME and orchestrating the cancer immune responses [33, 34, 35].

**FIGURE 1:**
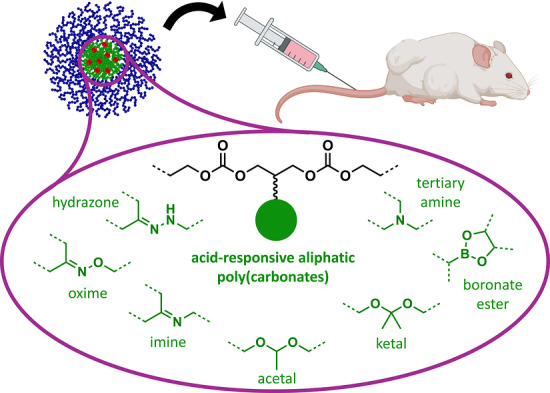
Design of acid‑responsive aliphatic polycarbonates enabling controlled micellar release for anti‑tumor drug delivery (created with BioRender).

Typical acid labile functionalities are oximes, imines, hydrazones, boronate ester, acetals or ketals, as well as nanocarrier unfolding via ionizable groups such as tertiary amines (Figure 1) [36, 37, 38, 39, 40]. Among these chemical motifs hydrazones, oximes, and imines (Schiff bases) undergo cleavage under acidic conditions of typically pH 4.5–6.5, a property that makes them well‐suited for endosomal‐triggered drug release. While imines generally hydrolyze the fastest, hydrazones provide a more controlled and tunable balance between stability and lability, whereas oximes, by contrast, are markedly more resistant to hydrolysis at neutral pH and require stronger acidic conditions for cleavage, offering excellent protection against premature drug release [36, 37, 41, 42, 43, 44]. Acetals and ketals also degrade through acid‐catalyzed mechanisms, and their hydrolysis rates can be engineered across several orders of magnitude [44, 45, 46]. This versatility has made them valuable motifs for constructing degradable polymer carriers; in particular, ketals have emerged as especially promising for intracellular nanocarrier breakdown and controlled drug delivery [47, 48, 49, 50, 51, 52, 53, 54, 55]. Boronate esters introduce an additional layer of responsiveness: they are cleaved not only by acidic pH but also by oxidative species, enabling dual‐trigger systems that exploit features of the tumor microenvironment such as elevated reactive oxygen species levels [38, 56, 57]. Polymers containing tertiary amines respond to acidic conditions not by cleavage but by protonation. This protonation can induce the so‐called proton‐sponge effect, promoting endosomal swelling and/or membrane destabilization and thereby facilitating endosomal escape [58, 59, 60]. Taken together, these functional groups span a wide landscape of acid lability and mechanistic behavior, enabling fine‐tuning of drug‐release profiles in response to physiological pH gradients, as summarized by Table 1. However, when comparing their direct pH responsiveness, it is crucial to recognize the underlying differences in cleavage mechanisms, environmental sensitivities, substituent effects, and aqueous accessibility. Even when different motifs operate nominally within similar pH ranges, their individual performance may diverge substantially, making direct one‐to‐one comparisons inherently limited.

**TABLE 1:** Summary of Common Acid‐Responsive Chemical Motifs Used in Stimuli‐Responsive Nanocarriers.

chemical motif	typical pH‐responsive range	relative degradation rate	mechanistic notes	considerations for drug delivery	ref.
**hydrazone**	4.5 – 6.0	moderate; half‐lives range from minutes to hours depending on substituents	proton catalyzed hydrolysis into aldehydes/ ketones and hydrazines/ hydrazides, accelerated by electron‐withdrawing groups	widely used for drug–polymer or drug–lipid conjugation; good selectivity for endosomal pH	[36, 41]
**oxime**	4.0 – 5.5	slower than imines/ hydrazones	oxime C=N bond is more stable; requires lower pH for efficient cleavage into aldehydes/ ketones and O‐substituted hydroxyl amines	oximes are markedly more stable than hydrazones, with hydrolysis rates at neutral pH rate ∼1000‐fold lower than for hydrazones, whereas both accelerate under mild acidity	[36, 43]
**imine (Schiff base)**	5.5 – 6.5	fast to moderate	proton catalyzed hydrolysis of C=N already under mildly acidic environments into aldehydes/ ketones and amines	useful for rapid drug release, but stability at physiological pH can vary with steric/electronic structure	[37, 44]
**acetal/ ketal**	4.0 – 6.0	fast to slow depending on structure	acid‐catalyzed cleavage to aldehydes/ ketones and two alcohols	widely applied in crosslinkers, polymer side chains and backbones; tunable hydrolysis kinetics over >6 orders of magnitude via substituent design	[39, 45, 46]
**boronate ester**	5.0 – 7.0 (also ROS responsive)	rapid hydrolysis (near pH 5 –6)	acid‐catalyzed cleavage by protonation of boronate ester, making the boron center more electrophilic and susceptible to water	dual pH and also ROS sensitivity; often applied for tumor microenvironment targeting	[38, 56, 57]
**tertiary amines**	5.0 – 7.0 (protonation)	structural swelling/ unfolding increases rapidly with protonation	no bond cleavage but pK_A_‐controlled protonation of the amine, often referred to as proton sponge effect leading to endosomal swelling and/ or membrane destabilization	enables endosomal escape and tunable pK_A_ via structural modification (currently most successfully applied in many lipid nanoparticle systems)	[58, 59, 60]

Especially in the context of cancer immunotherapy, the strategy of acid labile functionalities can be employed to deliver immunomodulatory drugs directly into the TME and its associated immune cells, thereby, activating innate immune processes and subsequently stimulating adaptive immunity, ultimately restoring the body's ability of recognizing and eliminating cancer cells [61]. Therefore, the mildly reduced pH value is of particular interest as stimulus, and respective carriers can easily be adapted to address multiple targets.

Among the diverse polymer carriers investigated for drug delivery, aliphatic carbonates have recently gained prominence as highly promising candidates [62]. Their inherent hydrolytic degradability, coupled with excellent biocompatibility, underscores their suitability for biomedical use and supports their prospective translation into clinical applications [63, 64, 65]. Carbonate units have previously been incorporated into degradable polymer systems, either as pendent functionalities located in the side chains [66, 67] or, more importantly, as integral structural motifs within the backbones of aliphatic polycarbonates [68, 69, 70]. Beyond reversible step‐growth polymerization [71] they can be accessed most successfully by acid‐ or base‐catalyzed ring opening polymerization strategies of six‐membered trimethylene carbonate monomers under controlled living polymerization conditions affording high control over the degree of polymerization and end group modification [62]. Consequently, amphiphilic block copolymers derived from such polycarbonates can be generated that readily self‐assemble into nanostructured formulations (micelles, polymers or nanogels) and applied as carrier systems across a range of therapeutic contexts [72, 73, 74, 75, 76, 77, 78, 79, 80, 81, 82, 83, 84]. Furthermore, it is highly beneficial to combine aliphatic polycarbonates with acid labile functionalities to constrain their utility in applications requiring precise monitoring of carrier behavior during drug delivery. While monomers with acid‐sensitive functionalities can be directly copolymerized by base‐catalyzed ring‐opening polymerization conditions, the acid‐labile groups can also be introduced afterwards by facile post‐polymerization modification strategies that do not harm the backbone integrity [85].

In the following, the benefits of micellar drug formulations combining the appealing biodegradability of aliphatic poly(carbonate)s and the universal acid‐responsive trigger mechanism are reviewed with an emphasis on tumor therapy (Figure 1). A differentiation is made between systems in which an acid‐labile group is used for the reversible binding of a pharmaceutically active compound and systems in which micelle degradability is provided by acid‐responsive mechanisms. Both groups are classified according to the chemical functionalities required for acid‐responsiveness.

## 2 Acid Responsive Drug Conjugation

**FIGURE 2:**
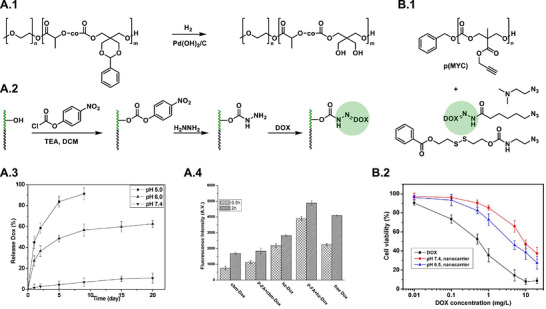
(A) Acid responsive DOX‐conjugation via hydrazone linkage reported by Hu et al. Deprotection of acetals gave access to hydroxy functionalities along the carbonate block that were used for further functionalization (A.1). An active‐carbonate approach enabled the covalent attachment of DOX via hydrazone linkage (A.2). Micelles formulated from DOX‐labeled block copolymers revealed pH‐dependent drug release (A.3) and superior uptake by folic receptor positive SKOV‐3 cells when co‐formulated with folic acid (FA) labeled polymers (A.4). (Reprinted (adapted) with permission from ref [86, 87]. Copyright 2010 American Chemical Society.) (B) Functionalizable poly(carbonate) p(MYC) reported by Yu et al. with propargyl side chains for post‐polymerization functionalization with azide labeled molecules. The approach was used to attach ionizable amines, side groups with DOX linked via hydrazone functionality and side groups including reductive responsive disulfides (B.1). Co‐formulation with amphiphilic block copolymers gave access to dual‐responsive micelles with pronounced cytotoxicity toward HeLa cells (B.2). (Reprinted (adapted) with permission from ref [88]. Copyright 2017 Elsevier.).

Since certain drugs, such as the chemotherapeutics doxorubicin (DOX) contain ketone functionalities, it is a straightforward strategy to conjugate these drugs via oximes, hydrazones or Schiff bases to a carrier. This strategy reduces drug leakage by the stable covalent bond at physiological pH but facilitates the release within mild acidic environments. In a pioneering work, Hu et al. reported on the design of amphiphilic block copolymers based on hydrophilic PEG and a hydrophobic poly(lactide‐*co*‐carbonate) block [86]. Along the carbonate repeating units hydroxy groups were protected as acetal benzyl acetal groups that could be removed upon catalytic hydrogenation (Figure 2A.1) [87]. Following an activated carbonate approach, hydrazine was attached to the hydroxy groups and in a second step used for conjugating DOX via a hydrazone linkage (Figure 2A.2). The obtained block copolymer self‐assembled into polymeric micelles with sizes of 70–100 nm and pronounced DOX release was demonstrated at pH 5.00 while distinctively less DOX was released at pH 7.4 (Figure 2A.3). The authors co‐formulated their amphiphilic block copolymers with folic acid (FA) labeled polymers to target folic receptor positive SKOV‐3 cells. They observed pronounced cell uptake that outperformed formulations without FA labeling and a non‐responsive reference carrying DOX conjugate by a carbamate linker (Figure 2A.4). Finally, DOX accumulated in the cell nuclei, where it induced significant cytotoxic effects.

By a similar approach Jiang et al. grafted DOX via hydrazone linkage to a block copolymer based on PEG and a carbonate block with vinyl side groups [89]. The latter were treated with mCPBA to obtain the epoxides, which were then ring opened. Similar as before, DOX was then attached covalently via a hydrazone linkage. Although increased DOX release was observed at pH 5.0, substantial drug leakage was still present at physiological pH. However, pronounced cell uptake was demonstrated with cytotoxic activity in HeLa cells. Wang et al. synthesized a poly(carbonate) with vinyl side groups, which they used for the functionalization with ethane‐1,2‐ditiol [90]. The obtained thiol groups were used for attachment of phosphorylcholine side groups that provided a hydrophilic domain and DOX that was conjugated via hydrazone functionality to a maleimide linker. The graft polymer self‐assembled into polymeric micelles that revealed a moderate pH‐responsiveness between pH 7.4 and 5.0 and showed cytotoxic efficacy against HeLa cells. Yu and coworkers synthesized a functional poly(carbonate) derived from 5‐methyl‐5‐propargyloxycarbonyl‐1,3‐dioxane‐2‐one (MYC) [88]. The propargyl side group was applied to a post‐polymerization modification with azide functionalized moieties via Cu‐catalyzed click chemistry (Figure 2B.1). The introduction of acid‐ and reductive‐responsive side groups enabled the formation of a dual‐degradable polymer. Co‐formulation with amphiphilic block copolymers gave access to micelles with tunable sizes. The dual‐responsive mechanism was demonstrated in vitro revealing pronounced DOX release at pH 5.0 and upon co‐stimulation with GSH. In addition, DOX related cytotoxicity toward HeLa cells was increased at pH 6.5 compared to physiological conditions (Figure 2B.2).

**FIGURE 3:**
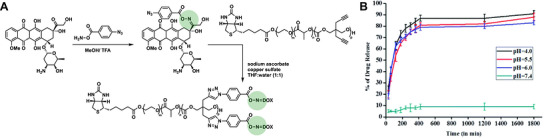
Acid responsive DOX‐conjugation via oxime linkage reported by Ganivada et al. (A) The polymer was composed of a hydrophilic PEG block and a hydrophobic poly(lactide‑co‑carbonate) block containing acetylene groups for covalent coupling by click chemistry. DOX was linked to the polymer backbone via an oxime group attached to an azide linker. Besides, biotin was added to the PEG chain end for active targeting. (B) In water, the conjugates self‑assembled into micelles that remained stable at physiological pH but released DOX efficiently under acidic conditions (pH 6.0). (Reprinted (adapted) with permission from ref [91]. Copyright 2016 The Royal Society of Chemistry).

Ganivada et al. reported DOX conjugation to macromolecular carriers via an oxime functionality as pH‐responsive group (Figure 3) [91]. For their approach, they synthesized block copolymers with a hydrophilic PEG block and a hydrophobic poly(lactide‐*co*‐carbonate) block. Acetylene side groups along the carbonate repeating units were used for covalent attachment of azide‐labeled molecules. Using this approach, DOX was conjugated to the polymer via an oxime‐azide linker (Figure 3A). In addition, biotin was conjugated to the PEG chain end as an active targeting ligand. In aqueous medium, these polymers self‐assembled into polymeric micelles that revealed distinct acid‐responsive DOX release at pH 6.0 while remaining stable at physiological conditions (Figure 3B). The formulation successfully reduced HeLa cell proliferation in vitro. However, a potential benefit derived from the biotin ligand was not demonstrated.

**FIGURE 4:**
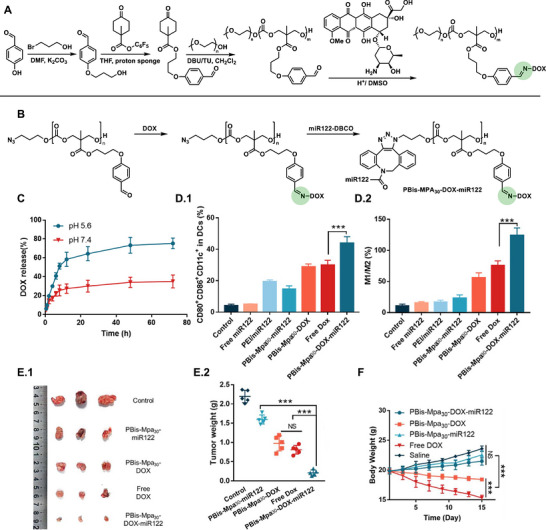
Dual‐responsive Nanocarrier delivery approach reported by Ke et al., and Jiang et al. (A) An aldehyde‑functionalized cyclic carbonate monomer was synthesized by Ke et al. and polymerized onto mPEG macroinitiators to form amphiphilic block copolymer, where. DOX was attached via its amine group through imine (Schiff base) formation. (Reprinted (adapted) with permission from ref [74]. Copyright 2014 The Royal Society of Chemistry.). (B) Jiang et al. followed this conjugation strategy but with azide end group functionalized poly(carbonate)s bearing benzaldehyde side groups for imine (Schiff base) DOX ligation. The azide end group was applied for conjugating liver‐specific miR122. (C) Acid sensitive DOX release. (D) In vitro immune cell activation showing the upregulation of CD80/86 surface maturation markers on DCs (D.1) and the repolarization of M2‐polarized macrophages to anti‐tumoral M1‐polarized macrophages (D.2). (PEI referees to branched polyethyleneimine as non‐responsive reference system.) (E) In vivo anti‐tumor efficacy study in Hepa1‐6 tumor‐bearing mice provided reduced tumor sizes (E.1) and tumor weights (E.2) after 14 d of treatment. (F) The time‐dependent changes of body weight. (Reprinted (adapted) with permission from ref [94]. Copyright 2024 Elsevier.).

Ke et al. demonstrated acid‐responsive DOX release using a Schiff base (imine) conjugation strategy (Figure 4A) [74]. For this purpose, they synthesized a cyclic carbonate monomer with aldehyde functionality along the side chain which they polymerized using methoxy‐PEG (mPEG) as macroinitiator. The obtained amphiphilic block copolymer was used to link DOX via its amine functionality under the formation of an imine. Although pronounced DOX release was already observed at pH 7.4, an accelerated increase could be provoked by reducing the pH to 5.0. By comparing the cytotoxicity of the formulation and free DOX toward MCF‐7 cells and DOX resistant MCF‐7/Adr cells, the authors emphasized the benefit of formulating DOX and overcoming evading mechanisms that cells have developed for drug resistance. While distinctively reduced cytotoxicity was observed for free DOX against MCF‐7/Adr cells, the activity of formulated DOX was restored to an extent that was observed in case of non‐resistant MCF‐7 cells. Kalva et al. synthesized amphiphilic block copolymers from mPEG and a carbonate monomer with *o*‐nitrobenzyl (ONB) esters along the side group [92]. In addition, acetylene moieties in the carbonate blocks side chain served for conjugating azide‐labeled molecules via Cu‐catalyzed click chemistry. Following this approach, they incorporated an aldehyde into the carbonate blocks which was used to ligate DOX as Schiff base. Formulating these polymers in aqueous media resulted in micelles with diameters of 20–40 nm that not only provided acid responsive DOX release but also UV‐stimulated micelle degradation due to photocleavage of the ONB esters. In vitro studies with colon cancer cells (CT26) revealed pronounced cell uptake of the formulations and a sustained drug release compared to free DOX. The same authors reported a carbonate homopolymer which again was functionalized via click reactions to obtain functional brush‐like polymers [93]. PEG domains were grafted onto the polymer and, similar to the previously reported structures, DOX was conjugated as Schiff base. Since ring opening polymerization (ROP) of the carbonate monomer was initiated by tetraphenylethylene methanol (TPE‐OH), a fluorescent end group was incorporated providing aggregation‐induced emission for tracking micelles in vitro. The formulations showed increased DOX release under acidic conditions and substantial cell uptake and cytotoxicity against CT26 cells. Finally, Jiang et al. used the same carbonate monomer introduced by Ke et al. to obtain carbonate homopolymers with benzaldehyde moieties along the side chain for DOX ligation (Figure 4B) [94]. In addition, they conjugated a liver‐specific miRNA miR122 modified with dibenzocyclooctyne (DBCO) groups to the azide functionalized chain end via Cu‐free strain promoted alkyne‐azide click reaction. Besides triggering anti‐tumor immune responses by enhancing innate immunity, the miR122 served as hydrophilic domain, resulting in the self‐assembly of the structures into spherical nucleic acid (SNA) nanoparticles. Release studies demonstrated a pH sensitive DOX liberation (Figure 4C) and sustained RNA stability in the presence of RNase A at physiological pH, while under acidic condition (pH = 5) miR122 rapidly degraded due to the loss of its nanoparticular structure affording fast miR122 degradation by RNase A. In vitro immune response studies demonstrated the synergistic efficacy of the DOX‐miR122 co‐formulation triggering an upregulation of CD80/86 surface maturation markers on dendritic cells (DCs, Figure 4D.1) and the repolarization of M2‐polarized macrophages to an M1‐like anti‐tumoral phenotype (Figure 4D.2). Finally, the in vivo anti‐tumor potency was tested with Hepa1‐6 tumor‐bearing C57BL/6J mice. The administration of the DOX‐miR122 co‐formulation induced remarkable tumor growth suppression and outperformed free DOX and other formulations with the individual components (Figure 4E). In addition, body weight loss resulting from free DOX administration was prevented by formulating the drugs, underlining the reduction of side effects associated with systemic drug administration (Figure 4F).

Another acid degradable functionality that is commonly used to reversibly bind drugs are boronate esters. Although, they show excellent pH responsive properties, their use is limited as they are only compatible with diol‐ or catechol‐containing compounds. Aguirre‐Chagala et al. developed amphiphilic block copolymers with the diol‐containing drug capecitabine (CAPE) attached to the carbonate block via boronate esters [95]. For this purpose, they synthesize the pinacol‐protected precursor polymer and deprotected it, using an excess of phenyl‐1,4‐diboronic acid [96]. Subsequently, they installed CAPE with a degree of functionalization of 75%. The obtained polymers were able to self‐assemble into spherical nanoparticles and showed substantial stability under physiological conditions while rapidly liberating the drug at acidic pH values. Liu and coworkers incorporated catechol functionalities into the side groups of poly(carbonate) blocks to obtain functionalizable block copolymers [97]. Afterwards, bortezomib, an anti‐cancer drug containing a phenylboronic acid group, was conjugated to the poly(carbonate) blocks side groups via pH‐responsive boronate ester functionality. In addition, an acetal functionality installed between PEG and the poly(carbonate) block facilitated acidic particle degradation and drug release. The formulation revealed a strong anti‐tumor activity in a human breast cancer BT‐474 xenograft mouse model that outperformed the free drug and reduced side effects. In a similar approach the same group used amphiphilic block copolymers with catechol functionalities along their side groups to reversibly bind DOX via pH‐sensitive *p*‐quinoneimine functionalities [98]. Co‐formulation with imidazole side chain containing polymers yielded stable particles showing no premature drug leakage, but rapid drug release under acidic conditions. The formulation revealed superior anti‐cancer activity compared to free DOX without causing systemic toxicity in a mouse breast cancer model. The same group conjugated apomorphine (AMP), a drug used in the treatment of Parkinson's disease, to block copolymers via boronate ester functionality [99]. This formulation effectively protected the oxidation sensitive drug from the environment, avoiding the formation of toxic oxidation products. Under acidic conditions, however, the drug was rapidly liberated.

Altogether, acid‑responsive drug conjugation onto aliphatic polycarbonates represents a powerful strategy for achieving controlled and tumor‑selective delivery of therapeutics such as doxorubicin. Using acid‑labile groups like hydrazones, oximes, imines, or boronate esters enables the creation of diverse polymer architectures that are stable at physiological pH yet release their payloads efficiently in the mildly acidic environments of tumors and intracellular compartments. Different conjugation chemistries, however, can influence the sensitivity of the pH trigger, and variations in tumor microenvironments may further affect drug release. Nonetheless, these functionalized polycarbonates can be incorporated into amphiphilic block copolymers that self‑assemble into micellar nanoparticles with tunable sizes, multifunctionality, and options for active targeting or combination therapy. Importantly, the carriers themselves should also contain acid‑responsive motifs to promote accelerated degradation and clearance from the body.

## 3 Acid Responsive Carrier Decomposition

**FIGURE 5:**
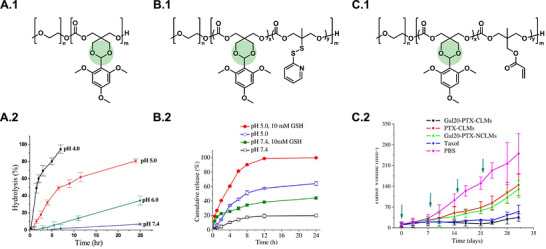
Nanocarrier design using TMBPEC as pH‐responsive hydrophobic poly(carbonate) block. (A) In a pioneering work Chen et al. reported on PEG‐*b*‐p(TMBPEC) block copolymers (A.1) that self‐assembled into polymeric micelles that were used to encapsulate DOX and release it in a pH responsive manner (A.2). (Reprinted (adapted) with permission from ref [100]. Copyright 2009 American Chemical Society.) (B) The authors extended the carrier system by incorporating activated disulfide functionalities into the poly(carbonate) block (B.1). These disulfides were used for micelle core crosslinking. Encapsulated DOX was released from the micelles in a dual‐responsive mechanism upon acidification and/or under elevated GSH concentrations (B.2). (Reprinted (adapted) with permission from ref [103]. Copyright 2015 Elsevier.) (C) The integration of acryloyl side groups allowed for reversible and irreversible micelle crosslinking (C.1). (Reprinted (adapted) with permission from ref [104]. Copyright 2012 Elsevier.) The co‐formulation with galactose labeled polymers supported active targeting of the asialoglycoprotein receptor (ASGP‐R) overexpressing on human hepatoblastoma cells (HepG2) and resulting in an improved in vivo anti‐tumor activity (C.2). (Reprinted (adapted) with permission from ref [105]. Copyright 2014 Elsevier.).

Early attempts for the design of acid‐degradable nanocarriers based on aliphatic poly(carbonate)s were performed by Chen et al. by designing amphiphilic block copolymers with acid‐responsive acetal moieties in the hydrophobic side chains [100]. Starting from pentaerythritol they synthesized two cyclic carbonate monomers (2,4,6‐trimethoxybenzylidene‐pentaerythritol carbonate (TMBPEC) and mono‐4‐methoxybenzylidene‐pentaerythritol carbonate (MBPEC)). Increased electron donation of the trimethoxybenzylidene moiety supported accelerated acetal hydrolysis of TMBPEC under acidic conditions, making it a promising candidate for the design of pH sensitive carriers. ROP of the monomer with a mPEG macroinitiator yielded amphiphilic block copolymers (Figure 5A.1) that self‐assembled into nanometer‐sized micelles with excellent pH‐responsive properties. Nile Red release studies demonstrated amplified cargo release under acidic condensation (Figure 5A.2), which could be reproduced for the release of encapsulated DOX and paclitaxel (PTX). By varying the carbonate block length, the same authors obtained polymeric vesicles, that demonstrated comparable pH responsiveness and allowed for the delivery of hydrophilic cargos [101]. The authors further developed this carrier by designing dual‐responsive micelles [102]. The incorporation of a disulfide bridge between the PEG block and the poly(carbonate) block enabled reductive micelle decomposition in addition to the acid‐triggered carbonate block hydrophilization. The design facilitated endosomal micelle swelling and drug release, followed by reductive polymer cleavage in the cytosol, triggered by the elevated GSH levels characteristic for cancer cells. The authors demonstrated DOX release upon eliciting the respective degradation profile and improved drug liberation in case of dual stimulation. In a similar approach, the authors designed a PEG‐*b*‐poly(carbonate) block copolymer with a mixed poly(carbonate) block obtained by copolymerizing TMBPEC and PDSC (pyridyl disulfide cyclic carbonate), a carbonate monomer with activated disulfide side groups for post‐polymerization modification (Figure 5B.1) [103].

After formulating polymeric micelles, the activated disulfides were used to crosslink the micelles cores by dithioerythritol (DTT). Encapsulated DOX was efficiently retained by the intact micelles while stimulation with 10 mmol/L GSH at pH 5 induced complete drug release (Figure 5B.2). In vitro experiments revealed an increased anti‐tumor activity of these formulations compared to irreversibly crosslinked micelles. The authors further modified their delivery platform by synthesizing PEG‐*b*‐poly(carbonate) amphiphiles with a carbonate block comprising TMBPEC and carbonate repeating unity with acryloyl side groups (Figure 5C.1) [104]. The latter were used for reversible or irreversible crosslinking of the micelles’ cores to improve micellar stability. Despite the crosslinking, the micelles retained their pH responsive drug release properties as demonstrated for PTX delivery into MCF‐7 and RAW264.7 cells. The analog approach was combined with an active targeting strategy [105]. For this purpose, micelles were co‐formulated with amphiphilic polymers that were decorated with a galactose unit at their hydrophilic chain end. The PTX loaded formulations showed improved receptor‐mediated endocytosis by asialoglycoprotein receptor (ASGP‐R) over‐expressing human hepatoblastoma cells (HepG2). The hepatoma‐targeting anti‐tumor efficacy of the formulation was further evaluated in vivo by treating SMMC‐7721 bearing nude mice. Improved tumor accumulation and significantly reduced tumor volumes were observed after 32 d (Figure 5C.2). Qiu et al. used basic PEG‐*b*‐p(TMBPEC) block copolymers for DOX encapsulation and conjugated the active targeting ligand cRGDyK to the nanoparticles surfaces [106]. Although the pH‐sensitivity was relatively moderate, animal studies confirmed enhanced tumor accumulation of the nanoparticle formulations, which effectively inhibited tumor growth while simultaneously reducing side effects. Yi et al. co‐polymerized TMBPEC together with 5‐methyl‐5‐propargyl‐1,3‐dioxan‐2‐one (MPMC) to an mPEG block yielding amphiphilic block copolymers with acid‐responsive acetal side groups and functionalizable acetylene moieties inside the poly(carbonate) block [107]. They formulated polymeric micelles which they stabilized by crosslinking the acetylene groups either with 1,6‐diazidohexane or with bis(azidoethyl)disulfide to obtain dual‐responsive nanoparticles. DOX delivery into drug resistant MCF‐7/ADR cells demonstrated the effective shielding of the drug overcoming the delivery barrier. Furthermore, the dual‐sensitive micelles revealed enhance apoptosis of these cells presumably due to DOX release as a result of the mildly acidic endosomal environment and elevated GSH levels in the cytoplasm.

**FIGURE 6:**
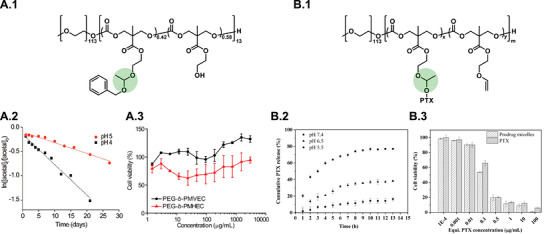
Amphiphilic block copolymers with functionalizable vinyl ether side groups along the carbonate blocks repeating units for post‐polymerization modification. (A) Cho et al. reported a novel carbonate monomer with vinyl ether side groups and synthesized amphiphilic block copolymers via ROP using mPEG as macroinitiator. Functionalization of the side groups allowed partial acetal formation (A.1). The obtained polymers provided pH‐responsive acetal hydrolysis (A.2) and were characterized by excellent biocompatibility (A.3). (Reprinted (adapted) with permission from ref [110]. Copyright 2018 American Chemical Society.) (B) Zhou et al. utilized this approach to synthesize PEG‐*b*‐poly(carbonate) amphiphiles, which were subsequently functionalized with PTX (B.1). The obtained structures revealed acid responsive PTX release (B.2) and pronounced cytotoxicity against A549 cells (B.3). (Reprinted (adapted) with permission from ref [111]. Copyright 2020 Informa UK Limited, trading as Taylor and Francis Group.).

Arno et al. used similar acetal‐functionalized poly(carbonate)s to obtain homopolymers with norbornene moieties [108]. Post‐polymerization modification of the norbornenes’ double bound allowed for versatile functionalization with azides (1,3 dipolar cycloaddition), tetrazines (inverse electron demand Diels Alder reaction) and thiols (photoinduced radical thiol‐ene reaction). Using the thiol‐ene reaction they grafted hydrophilic PEG domains and hydrophilic aliphatic domains to the carbonate homopolymer. The obtained amphiphiles self‐assembled into nanometer‐sized micelles that were highly biocompatible and got internalized by human A549 and IMR‐ 90 cells. In a follow‐up paper, the authors used this platform to graft S‐(+)‐camptothecin (CPT), a topoisomerase I inhibitor widely used in cancer therapy, via 1,3 dipolar cycloaddition and obtained a pH responsive drug conjugate [109]. Hydrophilic PEG domains enabled the formation of these conjugates into micelles that effectively inhibited cell proliferation of cancer cell lines (A549 and PC3) while not affecting non‐cancerous cell lines (MR‐90 and HS792). Notably, this behavior was observed only for the formulated drug, while the soluble form exhibited pronounced cytotoxicity across all tested cell lines, highlighting the advantage of the acid‐responsive nanoformulation. Cho and coworkers developed a functionalizable carbonate monomer with vinyl ether side group for subsequent functionalization [110]. They synthesized homopolymers and demonstrated their functionalizability by ligating alcohols and thiols to the vinyl ether functionalities under acid catalysis resulting in the formation of acetals and thioacetals (Figure 6A). Since the acidic catalysis facilitates hydrolysis in the presence of water, only partial functionalization was achieved while some side groups were transferred into hydroxy groups and some vinyl ethers remained unaffected. In addition, the authors also demonstrated the possibility to functionalize vinyl ether side groups via photoinduced thiol‐ene reaction resulting in a quantitative functionalization of all side groups. Finally, amphiphilic block copolymers were synthesized using mPEG as macroinitiator and partially modified to obtain acetal side groups (Figure 6A.1). The obtained polymers demonstrated, however, only decent pH responsiveness (Figure 6A.2).

**FIGURE 7:**
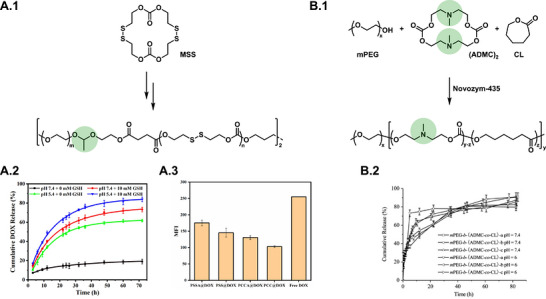
Acid‐responsive nanocarriers derived from macrocyclic carbonate monomers. (A) Qui et al. used the dithiocyclic macrocarbonate monomer (MSS) to obtain reductive‐responsive poly(carbonate)s which they modified with PEG by acid‐sensitive acetal group on both chain ends (A.1). Encapsulated DOX was released upon acidification and/or under reductive conditions (A.2). Additionally, the dual‐responsive formulation delivered DOX into the nuclei of HepG2 cells and outperformed control formulations with only one or no stimulus responsive functionality. (Reprinted (adapted) with permission from ref [115]. Copyright 2023 John Wiley and Sons.) (B) Macrocyclic carbonate monomers were already reported by Wang et al. who used (ADMC)_2_ to synthesize amphiphilic diblock terpolymers with ionizable tertiary amines along the polymer backbone (B.1). However, encapsulated ibuprofen was released pH‐independently (B.2). (Reprinted (adapted) with permission from ref [116]. Copyright 2012 Springer Nature.).

Self‐assembly in aqueous media yielded polymeric micelles that exhibited excellent cell compatibility (Figure 6A.3). Zhou et al. used this monomer to synthesize PEG‐*b*‐poly(carbonate) amphiphiles which they functionalized with PTX by the acetal linkage (Figure 6B.1) [111]. Micelles formulated from these conjugates demonstrated acid‐responsive PTX release (Figure 6B.2). The formulation was efficiently taken up by A549 cells and exhibited pronounced cytotoxicity, comparable to the free drug (Figure 6B.3). Yan et al. designed acid‐responsive block copolymers with cinnamaldehyde attached to the poly(carbonate) blocks side groups via acetals, and they formulated polymeric micelles with RSL3, a glutathione peroxidase 4 (GPX4) inhibitor, encapsulated into the micelles’ cores [112]. Both cinnamaldehyde, that serves as GSH‐depleting agent, and RSL3 aimed to reduce GSH levels in tumors, leading to lethal elevation of ROS produced by the tumors active metabolism. The authors demonstrated acid‐responsive cinnamaldehyde cleavage accompanied by micelle degradation and RSL3 release. In addition, they confirmed reduced intracellular GSH levels in 4T1 cells after incubating the cells with their formulation. Finally, excellent anti‐tumor activity was demonstrated in vitro and in vivo in a 4T1 cell xenotransplantation model, while side effects could be reduced. The same group reported a similar approach also with cinnamaldehyde linked to the poly(carbonate) block via acetal groups but with a simplified architecture [113]. Since high GSH levels in tumors contribute to drug resistant against anticancer drugs such as etoposide (ETS), they addressed this issue by encapsulating ETS into their cinnamaldehyde releasing carrier. The authors demonstrated effective depletion of intracellular GSH levels and an increased therapeutic effect in vitro. In addition, in vivo studies confirmed superior tumor suppression in 4T1 tumor‐bearing xenograft mice and reduced systemic activity of the formulation. Recently, Wang et al. expanded this concept and provided mixed‐micelle nanoparticles from acid‐responsive cinnamaldehyde acetal polycarbonates and polycarbonate contains Fe^3^^+^‐chelating carboxyl groups [114]. Within tumor cells, Fe^3^^+^ is reduced by GSH to Fe^2^^+^, simultaneously depleting GSH like cinnamaldehyde, and at the same time activating Fenton‐like reactions for ROS generation. Acidic pH also triggers micelle disassembly and release of encapsulated doxorubicin (DOX), which promotes apoptosis through DNA damage. By integrating H_2_O_2_ amplification, GSH depletion–mediated Fenton catalysis, and chemotherapy, the nanoplatform synergized improved anticancer therapy. Qiu et al. reported an alternative approach to obtain poly(carbonate) carriers with acid responsive acetal functionalities [115]. Derived from a dithiocyclic macrocarbonate monomer (MSS), they synthesized a carbonate homopolymer with disulfide bridges incorporated into the polymer backbone using 1,6‐hexanediol as initiator. In a subsequent reaction step, they functionalized both polymer end groups with PEG via an acetal group (Figure 7A.1). The obtained ABA triblock copolymers demonstrated dual responsiveness toward acidification and reductive treatments (Figure 7A.2). Encapsulated DOX was successfully delivered into HepG2 cells and could better enter the cell nuclei compared to nonresponsive carriers or such with only one stimuli‐responsive feature (Figure 7A.3). Unloaded micelles provided high biocompatibility while DOX delivery resulted in pronounced cytotoxicity.

The idea of synthesizing acid responsive poly(carbonate) carriers derived from macrocyclic carbonate monomers dates back to 2012 although acid responsiveness was then introduced by ionizable tertiary amines. Wang et al. used mPEG for the ring opening copolymerization of 6,14‐dimethyl‐1,3,9,11‐tetraoxa‐6,14‐diaza‐cyclohexadecane‐2,10‐dione ((ADMC)_2_) and *ε*‐caprolactone (CL) enzymatically catalyzed by Novozym‐438 (Figure 7B.1) [116]. The obtained diblock terpolymer self‐assembled into nanometer sized micelles (∼100 nm) that could swell under acidic pH conditions.

Although ibuprofen was successfully encapsulated, a drug release was already observed under physiological conditions and revealed no pH sensitivity (Figure 7B.2). However, this early work laid the foundation for numerous follow‐up studies. The same authors synthesized block copolymers by enzymatic ROP of (ADMC)_2_ and tetramethyl carbonate (TeME) [117]. Although drug release was again found to be pH‐independent, increased cytotoxicity of DOX‐loaded micelles against 293T cells was found compared to the free drug. Hu and colleagues picked up the idea and copolymerized (ADMC)_2_ and MSS to obtain a pH‐ and reductive‐dual‐responsive carrier [118]. Although the system suffered from multiple drawbacks, such as poor control over its polymerization and undefined encapsulation capacities, they sticked to the concept of macrocyclic carbonate monomers and developed a pH/ROS dual‐sensitive carrier [119]. For that purpose, they copolymerized (ADMC)_2_ and a selenide containing macrocyclic carbonate monomer using mPEG as macroinitiator and obtained amphiphilic diblock terpolymers. These polymers formed nanometer‐sized micelles with decent responsiveness to acidification and hydrogen peroxide as a result either of the protonation of the amine groups or hydrophilization of the selenide groups via oxidation. Within the tested concentration range, the carrier showed no cytotoxicity against EK293 cells and A549 cells. However, polymer concentrations were only tested to a level of 200 µg/mL. Finally, the authors extended their carrier platform with a selenide by using a diselenide containing macrocyclic carbonate monomer resulting in a triple responsive (GSH/ROS/acid) nanocarrier [120]. Encapsulated DOX was decently liberated upon stimulation with one or multiple triggers and in vitro investigations demonstrated pronounced cell uptake by A549 cells and significant cytotoxicity comparable to that of the free drug. In a similar approach Yang et al. enzymatically copolymerized dimethyl (selenobis(propane‐3,1‐diyl)) bis(carbonate) (DSPDC) and *N*‐propargyldiethanolamine to obtain polymers with ionizable tertiary amines and oxidizable selenide groups incorporated into the polymer backbone [121]. Azide‐functionalized PEG and TPE derivatives were grafted to the propargyl side groups via 1,3‐dipolar cycloaddition. Subsequently, the authors formulated polymeric micelles and used TPE‐related aggregation‐induced emission to evaluate micelles integrity. In addition, Nile Red (NR) loaded micelles were investigated revealing remarkable stability of the formulation under physiological conditions (pH = 7.4) but substantial micelle decomposition and NR release under mild acidic and oxidative conditions (pH = 6.8, 0.5 mm H_2_O_2_). Finally, the authors demonstrated particle uptake by U87 and HL‐7702 cells and confirmed excellent biocompatibility for their nanocarrier system.

**FIGURE 8:**
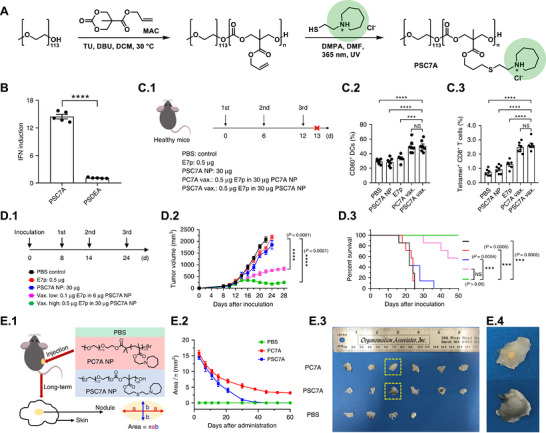
(A) Reaction scheme for the synthesis of the amphiphilic block copolymer with 7‐membered cyclic amine side groups (PSC7A). (B) STING activity of PSC7A was demonstrated in vitro and induced substantial IFN production in THP1‐ISG cells. In contrast the diethylamine‐derivative PSDEA did not activate STING. (C) In vivo performance of PSC7A co‐formulated with E7p compared reference samples upon subcutaneous administration at the tail base three times in 6 d intervals (C.1). The PSC7A nanovaccine increased the fraction of CD80^+^ dendritic cells (C.2) and tetramer^+^ CD8^+^ T cells (C.3). (D) Anti‐tumor activity of the PSC7A nanovaccine and reference samples after immunizing mice three times within 24 d (D.1). The PSC7A nanovaccine inhibited tumor growth (D.2) and prolonged survival of tumor‐bearing mice (D.3). (E) Side effects of the formulation were evaluated based on injection‐induced granulomatous inflammation. For this purpose, degradable PSC7A was compared with non‐degradable PC7A (E.1). While the nondegradable nanovaccine induced granulomatous inflammation that persisted over months, degradable PSC7A primed a transient acute inflammatory response followed by complete tissue healing (E.2–4). (Reprinted (adapted) with permission from ref [70]. Copyright 2020 Springer Nature.).

The concept of ionizable groups for pH dependent charge switch was used in further versatile nanoparticle designs. Based on 5‐methyl‐5‐benzyloxycarbonyl‐1,3‐dioxan‐2‐one (MTC‐OBn) Wang et al. synthesized block copolymers by ROP using mPEG as macroinitiator. Reductive benzyl ester cleavage via Pd/C catalyzed hydrogenation yielded poly(carbonate) blocks with carboxylic acid side groups [122]. In contrast to most other pH‐responsive carriers, these polymers were specifically designed to form stable micelles in their protonated state under acidic conditions. At neutral pH, deprotonation of the acid functionalities led to electrostatic repulsion triggering micelle disintegration. This approach aimed at enhancing the bioavailability of orally administered drugs by protecting them in the acidic environment of the stomach and enabling their release in the intestine. Guo and colleagues used the same repeating unit to exploit electrostatic interactions between carboxylic acid functionalities and amine groups of DOX for efficient drug encapsulation [123]. The formulations showed limited pH‐ and temperature‐responsive drug release but revealed good biocompatibility while preserving cytotoxicity of DOX in HeLa cells. Similarly, Li et al. synthesized block copolymers with vinyl side groups which they used to graft carboxylic acid functionalities onto the carbonate block via thiol‐ene reactions [124]. Again, they used electrostatic interactions to encapsulate DOX inside their polymeric micelles. The formulation showed limited pH responsiveness. However, DOX was successfully delivered to HeLa cells and demonstrated substantial cytotoxicity. Yu et al. used a similarly designed block copolymer and mixed it with tertiary amine functionalized poly(carbonate)s to obtain mixed micelles into which they encapsulated DOX [125]. Protonation of amins was supposed to induce pH‐responsive micelle degradation and drug release. While the unloaded carrier revealed great biocompatibility, the DOX‐loaded formulation efficiently inhibited proliferation of HepG2 cells. In a follow‐up study, the authors reported similar mixed micelles, further enhanced by a redox responsive trigger through incorporation of disulfide side groups [126]. In addition, an acid functionality was introduced via 1,2‐dicarboxylic‐cyclohexene and the obtained formulations revealed a much more sensitive pH‐responsiveness and distinct degradation under reductive conditions. DOX‐loaded micelles were effectively taken up by HeLa cells and revealed cytotoxic properties comparable to the free drug. Teo et al. grafted carboxylic acid functionalities via disulfide bridges to the carbonate block of amphiphilic bock copolymers to obtain pH/reductive dual‐responsive nanocarriers [127]. The authors demonstrated the potential of their carrier by DOX delivery to BT‐474 cells inducing efficient cytotoxicity. Finally, a substantial anti‐tumor efficacy was demonstrated in a BT‐474 human breast cancer xenograft mouse model. While tumor growth was effectively inhibited, no severe side effects were observed. Venkataraman et al. designed poly(carbonate)s with tertiary amine groups across the polymer backbone derived from functional eight‐membered *N*‐substituted aliphatic cyclic carbonate monomers [128]. The authors reported versatile monomers from which they obtained tertiary amine, secondary amine, and zwitterion residues incorporated along the polymer backbone. They also developed charge‐switchable pH‐responsive micelles which they used to encapsulate DOX and demonstrated pH‐responsive drug release. Xia et al. reported pH‐ and light‐sensitive nanoparticles [129]. Based on PEG‐*b*‐p(MYC), they grafted pH‐ and light‐responsive functionalities to the propargyl side groups via Cu(I)‐catalyzed alkyne‐azide cycloaddition. The pH‐responsiveness was introduced by incorporating tertiary amine groups and for the light sensitivity 4‐(4‐azidebutyloxy)‐4’‐trifluoromethoxy‐azobenzene (Azo‐N_3_) was additionally grafted onto the carbonate block. DOX‐loaded micelles revealed a pH‐responsive drug release that could further be enhanced by light‐induced disassembly. In vitro experiments confirmed the compatibility of the unloaded carrier and preserved cytotoxicity of the formulated DOX. Yunxia et al. synthesized poly(carbonate‐*co*‐ester) copolymers with primary amine side groups at the carbonate repeating units [130]. The authors used these amins as charge switchable groups and further grafted an active targeting ligand cRGD via a PEG spacer to the polymer. The obtained conjugates were used to formulate DOX‐loaded micelles. Although in vitro pH‐responsiveness was not very sensitive, in vivo experiments demonstrated effective tumor targeting in a nude mouse xenograft model of squamous cell carcinoma. Ray and colleagues synthesized PEG‐*b*‐poly(carbonate) block copolymers with tertiary amine side groups for charge switch and conjugated internalizing RGD (iRGD) as active targeting ligand to the PEG chain end [131]. Depending on the amine substituents, pH responsiveness at different pH levels was observed, which allowed for precise tunability of particle disassembly. As model drug, the authors encapsulated a combination chemotherapy used for pancreatic cancer (gemcitabine (GEM) and a Hedgehog inhibitor (GDC 0449)) and demonstrated targeted cell uptake in vitro and in vivo. The same authors also reported on 4‐amino‐4’‐dimethylaminoazobenzene (AZB, inspired by pH indicator methyl orange) and/or isoleucine (Ile) functionalized block copolymer which they used to encapsulate GEM [132]. The formulation showed pH‐dependent drug release and pronounced cytotoxicity against pancreatic ductal adenocarcinoma (PDAC) cells. Leong and coworkers used oppositely charged side groups to obtain stabilized micelles with high DOX loading capacities [133]. Based on 5‐methyl‐5‐allyloxycarbonyl‐1,3‐dioxan‐2‐one (MAC) they grafted urea derivatives or carboxylic acids to the carbonate blocks via thiol‐ene reaction. DOX loaded micelles revealed not only pH responsive drug release but also oxidative responsiveness as a result of oxidation of thioether groups to sulfoxides. Cell experiments demonstrated high anti‐tumor activity that was further confirmed in vivo. Wang et al. also used PEG‐*b*‐p(MAC) block copolymers to graft tertiary amines to the carbonate block via thiol‐ene reaction yielding degradable ultra‐pH sensitive (dUPS) polymers (Figure 8A) [70]. They screened multiple amine substitution patterns and found that a 7‐membered cyclic amine side group (PSC7A) can achieve activation of the stimulator of interferon genes (STING, Figure 8B). Based on these results, the authors generated a T‐cell vaccine by mixing PSC7A and the peptide antigen E7p (GQAEPDRAHYNIVTFCCKCD). The nanovaccine demonstrated robust DC and T cell activation (Figure 8C). Excellent anti‐cancer activity was observed by treating TC‐1 melanoma bearing mice resulting in the inhibition of tumor growth and improved survival rates (Figure 8D). Finally, the biocompatibility of the formulation was further emphasized by comparing the carbonate‐based vaccine with a non‐degradable reference (PC7A). While nondegradable nanoparticles induced granulomatous inflammation that persisted over months at the injection site, degradable PSC7A primed a transient acute inflammatory response followed by polymer degradation and complete tissue healing (Figure 8E).

The same 7‐membered cyclic amine side group was used by Guo and coworkers. Following a different functionalization strategy, they first polymerized MTC‐OBn, followed by reductive benzyl ester cleavage [134]. The obtained carboxylic acid groups were used for functionalization via Steglich esterification introducing the 7‐membered cyclic amine side group as ionizable moiety. In addition, a hexane‐1,6‐diamine linker was used that allowed for covalent DOX conjugation via Schiff base. The dual pH‐responsive conjugate self‐assembled into polymeric micelles that were further loaded with the chemotherapeutic agent lapatinib. The authors demonstrated pH‐responsive drug release, and the obtained micelles effectively inhibited the proliferation of 4T1 cells in vitro. Furthermore, a 4T1 xenograft‐bearing mice model was used to confirm anti‐tumor activity of the formulation, suppressing tumor growth and significantly decreasing pulmonary metastatic nodules, without significant systemic side effects. The authors also reported a nanocarrier with diisopropylamine side groups for pH‐responsive charge switch that was obtained following the same synthesis strategy [135]. Again, DOX was conjugated to the carbonate side group via Schiff base and the drug conjugate showed substantial 4T1 tumor cell inhibition in vitro.

A variant of the charge switchable carrier systems uses nucleobase pairs to stabilize micelles cores in non‐protonating environments. For instance, Kuang et al. coupled adenine and thymine to individual block copolymers and formulated mixed micelles that were stabilized by strong hydrogen interactions between the nucleobases [136]. They successfully encapsulated DOX and delivered it into MDA‐MB‐231 cells, inducing cytotoxicity comparable to that of the free drug. The same authors synthesized thymidine functionalized block copolymers and obtained stabilized micelles by co‐assembling these polymers with 9‐hexadecyladenine (A‐C16). Encapsulated DOX provided reduced leakage at pH 7.4 in case of increasing A‐C16 content, while accelerated release rates were observed at pH 5.0. In addition, DOX loaded micelles revealed pronounced cytotoxicity against MDA‐MB‐231 cells [137]. Cheng and colleagues used thymine‐functionalized amphiphilic block copolymers to encapsulate the anti‐cancer drug methotrexate (MTX) [138]. The 2,6‐diaminopyridine groups of MTX effectively interacted with thymidine resulting in high drug‐loading capacities and pH‐sensitive drug release. Cytotoxicity assays confirmed the biocompatibility of the thymidine‐functionalized carrier and demonstrated pronounced cytotoxicity of MTX‐loaded micelles against HeLa cells. The same group reported amphiphilic block copolymers with thymidine and carbonic acid moieties along the carbonate block [139]. Again, thymidine was used to bind MTX while acid functionalities were introduced to interact with amine groups of DOX. The formulation showed pH sensitive MTX‐ and DOX‐release. Confocal laser scanning microscopy revealed efficient cell uptake and showed DOX accumulation mainly in nuclei while MTX was found in the cytoplasm. Cell viability assays confirmed the pronounced cytotoxicity of the co‐formulated drugs.

He et al. used acid‐degradable β‐thiopropionate functionalities to introduce pH‐responsiveness into their nanocarrier systems. In contrast to other reported acid responsive functionalities, β‐thiopropionates are characterized by very slow hydrolyzation rates [140]. First, the authors synthesized PEG‐*b*‐poly(carbonate) block copolymers with acrylate functionalities along the carbonate block. Micelles formulated from these polymers were then crosslinked with 1,6‐hexanedithiol via thiol‐acrylate Michael addition. Release experiments of encapsulated coumarin 102 revealed accelerated release at pH 5.0 compared to physiological conditions. In a similar approach the authors crosslinked amphiphilic block copolymers with propargyl side groups using a dithiol crosslinker that incorporated the β‐thiopropionate functionality [141]. Due to the slow hydrolysis of the β‐thiopropionate, they achieved sustained release of encapsulated DOX. The unloaded carrier showed good biocompatibility while encapsulated DOX delivery resulted in excellent internalization and pronounced anticancer effectiveness in HeLa cells.

To obtain acid degradable nanocarriers Dominski et al. [142] used ketal functionalities in their biodegradable triblock terpolymer PEG‐*b*‐poly(carbonate)‐*b*‐oligo([R]‐3‐hydroxybutyrate). The polymers self‐assembled into micelles with diameters of ∼25 nm and swelled upon acid‐induced ketal hydrolysis resulting in the release of the encapsulated cargos. As model drugs, DOX and 8‐hydroxyquinoline glucose‐ and galactose conjugates were used. While unloaded micelles showed no significant cytotoxicity to different cell lines, glycoconjugates‐loaded micelles effectively inhibited the proliferation of MCF‐7 and HCT‐116 cells. In a follow‐up paper, the authors simplified the polymer architecture and synthesized amphiphilic PEG‐*b*‐poly(carbonate) block copolymers with acid‐degradable ketal side groups that self‐assembled into polymeric micelles [143]. Based on these micelles, supramolecular hydrogels were prepared by adding *α*‐cyclodextrin that formed host–guest complexed with the PEG chains. These hydrogels were designed to co‐deliver hydrophilic 8‐hydroxyquinoline glycoconjugate (8HQ‐Glu) and hydrophobic DOX. In vitro drug release studies confirmed the fast liberation of both compounds under acidic conditions. In addition, cell viability assays demonstrated synergistic anti‐cancer activity of the DOX/8HQ‐Glu co‐formulation. Lately, the amphiphilic PEG‐*b*‐poly(carbonate) block copolymers with acid‐degradable ketal side groups were also co‐formulated by Li et al. together with PEG‐*b*‐poly(carbonate) block copolymers containing hydrazone‐linked doxorubicin into acid‐responsive micellar nanocarriers [144]. On top, they also encapsulated the water‐insoluble epidermal growth factor receptor inhibitor erlotinib into the micellar core, thus, affording dual acid‐triggered drug release of erlotinib and doxorubicin. This resulted in vitro in a stronger inhibition of A549 cell proliferation than erlotinib or doxorubicin alone, and in vivo in reduction of a non‐small cell lung cancers, showcasing effective cancer therapy through sequence‐controlled, pH‐triggered co‐delivery of synergistic drug combinations.

**FIGURE 9:**
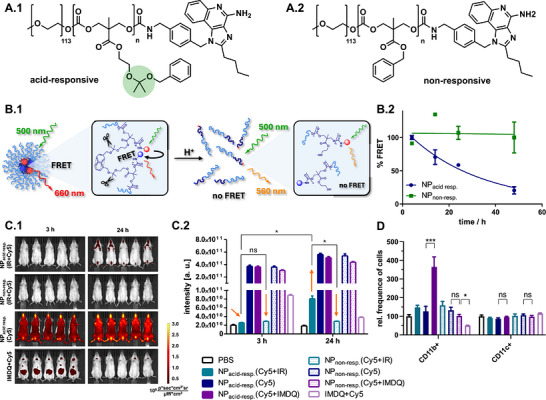
(A) Acid‐responsive amphiphilic polycarbonate block copolymers with benzyl ketal side groups (A.1) and non‐responsive benzyl esters (A.2), both end group functionalized with the TLR7/8 agonist IMDQ. (B) FRET‐based monitoring of Cy3‐ and Cy5‐end group labeled block copolymer unfolding (B.1), verified in vitro by flow cytometry of macrophages treated with acid‐responsive or the non‐responsive micelles (B.2). (C) In vivo FRET‐quencher system of Cy5‐ and IR‐end group labeled block copolymer micelles (C.1) demonstrating an exclusive unfolding for the Cy5+IR functionalized acid‐responsive micelles versus the non‐responsive micelles after 24 h (C.2). (D) Only the acid‐responsive amphiphilic polycarbonate block copolymers with IMDQ‐end group functionalization trigger highest recruitment of macrophages (Reprinted (adapted) with permission from ref [146]. Copyright 2025 Wiley.).

Recently, we could also reported on the design of a PEG‐*b*‐poly(carbonate) based nanocarrier with ketal functionalities along the carbonate blocks side groups (Figure 9A) [145]. In this case, the ketal functionality provided a micelle stabilizing benzyl group. Acidification induced rapid micelle disintegration as result of hydrophilization of the carbonate block and the loss of the stabilizing benzyl moiety. The unloaded micelles showed excellent biocompatibility. As model drug, the TLR7/8 agonist CL075 was hydrophobically encapsulated and successfully delivered into macrophages stimulating robust immune activating pathways. A cell viability assay revealed no significant cytotoxicity for both loaded and unloaded polymeric micelles. In a follow‐up study, the high precision control afforded by applied ring‐opening polymerization yielded to tailored end‐group functionalization, including the incorporation of fluorophores for Förster resonance energy transfer (FRET)‐based tracking of micelle integrity and disassembly (Figure 9B) [146]. Our studies could successfully demonstrate a distinct pH‐dependent degradation profile both in vitro and in vivo (Figure 9C). Comparative analyses with non‐responsive poly(carbonate)s confirm the superior performance of the acid‐sensitive architecture, which selectively disassembled under acidic conditions and, most importantly, accelerated polymer backbone cleavage. Additionally, the covalent attachment of a small‐molecule Toll‐like receptor 7/8 agonist facilitated its controlled activity, enhancing immune cell recruitment and localized cytokine secretion (Figure 9D). Collectively, these findings highlighted the promise of this biodegradable, acid‐responsive micellar nanocarrier as a precision delivery platform for safer and more effective immunotherapeutic interventions, particularly toward cancer immunotherapy.

In conclusion, acid‑responsive decomposition of aliphatic polycarbonate nanocarriers has emerged as a versatile and powerful strategy for controlled drug delivery. By incorporating acid‑labile moieties such as acetals, ketals, and ionizable tertiary amines, these polycarbonate architectures can undergo hydrophilization, charge switching, and ultimately backbone cleavage under the mildly acidic conditions characteristic of tumor microenvironments and intracellular compartments. These triggers enable precise carrier destabilization and promote rapid drug release once internalized by target cells. Additional layers of responsiveness including redox, ROS, and light sensitivity can further enhance the selectivity and efficiency of cargo liberation. Acid‑degradable polycarbonates have consistently demonstrated improved therapeutic outcomes, ranging from enhanced cytotoxicity in drug‑resistant cancer cells to synergistic effects in combination therapies and immunomodulatory applications. Altogether, these findings highlight acid‑responsive polycarbonate architectures as highly adaptable platforms capable of achieving controlled carrier degradation, minimized systemic toxicity, and optimized delivery of diverse therapeutics.

## 4 Conclusions and Outlook

The here collected reports on micellar nanocarriers with acid‐responsive degradation mechanisms based on biocompatible aliphatic poly(carbonate)s highlight the great potential of this approach for the future design of advanced drug delivery systems. Such systems offer significant promise for tumor therapy by leveraging the acidic tumor microenvironment toward achieving controlled drug release. We have distinguished two principal categories: (i) systems utilizing acid‐labile linkages for reversible conjugation of active pharmaceutical agents, and (ii) systems in which micelle disassembly or polymer backbone cleavage is triggered by acid‐responsive functionalities. Among these categories chemical motifs including oximes, imines, hydrazones, boronate esters, acetals, ketals, and tertiary amines confer the acid sensitivity. Toward clinical translation, one should take into account that the contributing chemical motifs should ideally not address toxicological concern. To that respect, despite their structural similarity to acetals, ketal functionalities have by far been less explored as pH‐responsive groups for acidic nanocarrier degradation, and may also become valuable strategies for reversible drug conjugation. This is particularly surprising given their superior pH sensitivity which results from the increased electron density at the ketal carbon and the presence of an additional electron‐donating aliphatic group [46, 147]. Moreover, the degradation products of ketals which are primarily ketones are generally more biocompatible and pose less toxicological concern than the aldehydes formed during acetal hydrolysis [148]. These advantages underscore the need for nanocarrier systems that integrate the favorable biocompatibility and biodegradability of poly(carbonate)s with the enhanced pH‐responsiveness of ketals to address unmet challenges in cancer (immuno‐)therapy.

## Conflicts of Interest

The authors declare no conflict of interest.

## Data Availability

The author has nothing to report.
